# Differential tolerance to nickel between *Dreissena polymorpha* and *Dreissena rostriformis bugensis* populations

**DOI:** 10.1038/s41598-018-19228-x

**Published:** 2018-01-15

**Authors:** Marine Potet, Laure Giambérini, Sandrine Pain-Devin, Fanny Louis, Carole Bertrand, Simon Devin

**Affiliations:** Université de Lorraine, CNRS UMR 7360, Laboratoire Interdisciplinaire des Environnements Continentaux (LIEC), Campus Bridoux, rue du Général Delestraint, 57070 Metz, France

## Abstract

Differential tolerance to stress is partly responsible for the heterogeneity of biomarker responses between populations of a sentinel species. Although currently used for freshwater biomonitoring, studies concerning inter-populational variability in tolerance to contaminants for the zebra mussel (*Dreissena polymorpha*) are scarce. Moreover, this well-known invader is currently replaced by another, the quagga mussel (*Dreissena rostriformis bugensis*). To evaluate the differential tolerance between dreissenids, several populations of both species were exposed to a high concentration of nickel. A LT_50_ (time when 50% of individuals were dead) was established for each population. Biomarker responses and internal nickel concentration were also measured, to link tolerance with physiological status. Results evidenced that *D. polymorpha* populations are more heterogeneous and more tolerant than *D. r. bugensis* ones. For *D. polymorpha* populations only, LT_50_ values were positively correlated with the nickel contamination *in situ*, with higher anti-oxidative defences and a higher Integrated Biomarker Response value in the field. Such findings may be explained by local adaptation and invasion dynamic within each species. The significance of this differential tolerance when using biomarker responses for biomonitoring purposes is thus discussed.

## Introduction

Aquatic ecosystems are the ultimate receptacle for many pollutants, and undergo many disturbances. To face this environmental problem, the European Water Framework Directive (WFD) was designed to evaluate, protect and restore aquatic systems^1^. Primarily, it mainly focused on chemical monitoring, along with the establishment of environmental quality standards, but the implementation of biological and ecological approaches quickly became essential in order to obtain a representative and integrative picture of the quality of a water body.

Among recommended biological tools^2^, biomarkers are defined as biochemical, physiological or behavioural parameters that can evidence exposure to and/or toxic effects caused by stressors^3,4^. They are currently used in biomonitoring to evidence the presence and effects of contaminants, and to understand their mode of toxic action at the individual and sub-individual scales^5–7^. The implementation of multi-biomarker approaches enables the understanding of different toxicity mechanisms triggered by multiple stressors existing in the field, that may with time lead to an overall adverse effect^8–10^.

Despite their usefulness, the deployment of biomarkers is still controversial^11–13^. Unlike the marine environment where some structuring is taking place^14,15^, there is no particularly reasoned strategy for the choice of biomarkers in freshwater biomonitoring, although several studies tend to promote their integration as monitoring tools^16–18^.

In this study, we investigated the role that differential tolerance to stress might have on biomarker responses. Indeed, tolerance emergence in several populations of a same species is not homogeneous, since each population will develop a tolerance for the parameters it faces in its environment. Hence, each population sets up mechanisms to deal with environmental stressors, and express different biological responses, which need to be evaluated if they are to be used in biomonitoring. This inter-populational variability in tolerance is in part responsible for the heterogeneity of biomarker responses among populations. Basal levels of biomarkers in the field are often different from one population to another, since each population is influenced by local environmental conditions and genetic characteristics^19,20^. Thus, to make a proper use of biomarkers, we need to know thoroughly the populations of sentinel organisms in order to be able to correctly interpret their responses to environmental changes.

The zebra mussel (*Dreissena polymorpha*) is a widespread invasive bivalve species, commonly found in freshwaters of the northern hemisphere^21^, and identified by the IUCN as one of the 100 of the World’s Worst Invasive Alien Species^22^. In these ecosystems, particularly exposed to contaminants and susceptible to biological invasions^23,24^, *D. polymorpha* has an important ecological role and populations can reach high densities^25,26^. It is a sedentary filter-feeder, able to tolerate a wide range of environmental contaminants. It is thus currently used in biomonitoring programs, to evaluate both the presence of contaminants through bioaccumulation measurements^27,28^, and their effects through the use of biomarkers, either in the field or in laboratory^29–31^. This species has recently been considered as the freshwater counterpart to *Mytilus edulis*^32^. To understand and correctly interpret biomarker responses measured in zebra mussels for biomonitoring, inter-populational variability in tolerance should be evaluated, but attention must also be paid to inter-specific variability. Indeed, in recent years, populations of *D. polymorpha* have progressively been replaced by a sister species, the quagga mussel (*Dreissena rostriformis bugensis*)^33–35^. It is thus important to wonder to what extent the new invasive can either be used in future biomonitoring programs.

The two species have been largely studied and exhibit different anatomical and physiological features (e.g. morphology^36^, feeding and growth^37,38^, reproduction^39^, environmental requirements^40–42^, bioaccumulation capacities^43^), but their tolerance to contaminants as well as their biological responses, have little been compared. Comparisons are often conducted between several populations of one species^9,44,45^ (inter-populational variability) or between the two species^46^ (inter-specific variability), but rarely on several populations of both species on a large geographical scale. To our knowledge, there is only one study dealing with both inter-populational and inter-specific differences in relation to pollution^10^. The others are generally relative to hypoxia^47^ or salinity^48^ effects and even heritability of heat tolerance^49^. However, these works are mainly focused on predicting the invasiveness of dreissenid species in contrasting habitats, not on their potential use as biomonitors.

**Figure 1:**
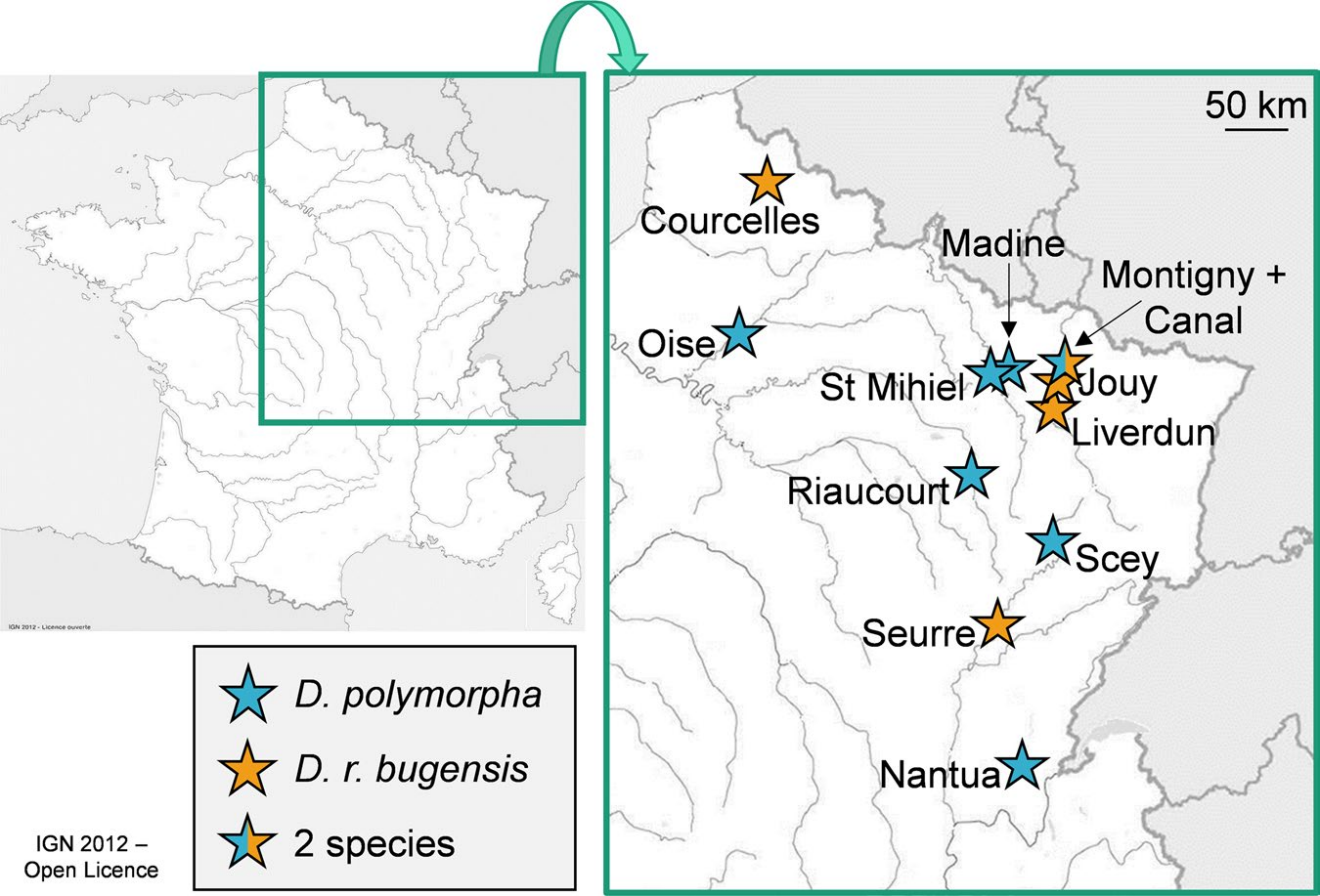
Sampling sites (modified from http://education.ign.fr/sites/all/files/carte_route_fluviale_5400k_13-2.jpg).

Our previous work^7^ has shown that *D. polymorpha* is more able to respond to contamination than his fellow, with a gradual biomarker response to low-dose Ni contamination. In the present study, we wanted to evaluate tolerance capacities in several populations of these species, along with their biomarker responses when facing an acute metal stress. We assumed that *D. polymorpha* populations, due to their better ability to cope with metal stress and to activate defence mechanisms, might be more tolerant than *D. r. bugensis* ones. In order to investigate this hypothesis, we sampled 13 populations of dreissenids (7 populations of *D. polymorpha* and 6 populations of *D. r. bugensis*) on 12 sites located in northeastern France (Fig. 1; for Montigny, the two species were simultaneously present). Organisms were brought to the laboratory and exposed to an acute concentration of nickel (Ni, 2.5 mg.L^−1^), in order to evaluate the LT_50_ (lethal time for 50% of the individuals) for each population. This measurement represents the time when half of the individuals died, which gives an indication of the tolerance to Ni in each population. Nickel has been selected in our study since it is one of the four priority metals defined by the WFD^50^, but ecotoxicological and environmentally relevant data for freshwater organisms are lacking. In order to explain the differential tolerance in tested populations, we first put LT_50_ in relationship with the sediment contamination on each site, to verify if the chronic exposure of populations was responsible for the observed differences in tolerance to Ni. Secondly, we measured Ni accumulation in organisms and a set of cellular biomarkers. Since nickel is known as a pro-oxidant agent in bivalves^51,52^, biomarkers of effects were preferentially selected, to evidence modifications in antioxidative mechanisms, energy metabolism and cellular damages.

## Results

### Differential Ni sensitivities in studied populations

**Figure 2:**
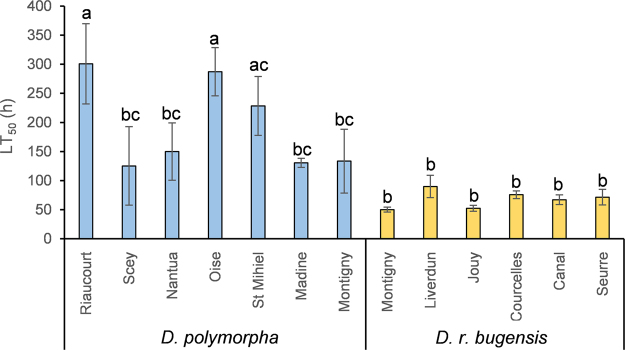
LT_50_ for each population exposed to nickel (2.5 mg.L^−1^), expressed as mean ± SD (n = 3). Differences between populations were evidenced through a Post Hoc Tukey test.

For a given population, LT_50_ values were calculated for each of the three replicates, computing mortality as a function of time. This means that we got three LT_50_ values per population (1 value for 5 individuals in one beaker). We observed differential tolerances to Ni in our populations (Fig. 2). No mortality was observed for control organisms, meaning that Ni-exposed mussels died because of the metal exposure, and not because of starvation or any other laboratory condition. Thus, only LT_50_ for Ni-exposed organisms are represented. Two-way ANOVA showed strong populational (*p* = 3.96·10^−5^, F = 6.59, df = 11, df_res_ = 26, n = 3) and species-specific (*p* = 1.94·10^−10^, F = 100.86, df = 1, df_res_ = 26, n = 3) effects. In a general way, *D. polymorpha* are more tolerant to Ni than *D. r. bugensis* populations. Inter-specific differences are very strong, with a mean LT_50_ for *D. polymorpha* (193.6 h ± 85.2) higher than for *D. r. bugensis* (67.8 h ± 16.7), and the highest value for *D. r. bugensis* never exceed the lowest value obtained for *D. polymorpha*. Responses are more heterogeneous for *D. polymorpha*, with strong inter-population differences, than for *D. r. bugensis* (no differences between populations). We should also note that on the site where both species are present (Montigny), *D. polymorpha* is more tolerant than its fellow is.

### Differences in sensitivity and local adaptation phenomena

Assuming that Ni contamination on a given site can increase the tolerance of the population living therein, a simple regression between mean LT_50_ for each population and Ni contamination in originating site was set up. No correlation was observed (*p* = 0.19, n = 13), which means that *in situ* Ni concentration has no effect on the LT_50_ observed in laboratory. However, when we looked at the two species separately, there was a correlation for *D. polymorpha* (*p* = 0.034, R² = 0.52, n = 7), suggesting that for this species, Ni tolerance depends on chronic exposure to Ni, while it was not observed for its conspecific (*p* = 0.37, R² = 0.03, n = 6).

**Figure 3:**
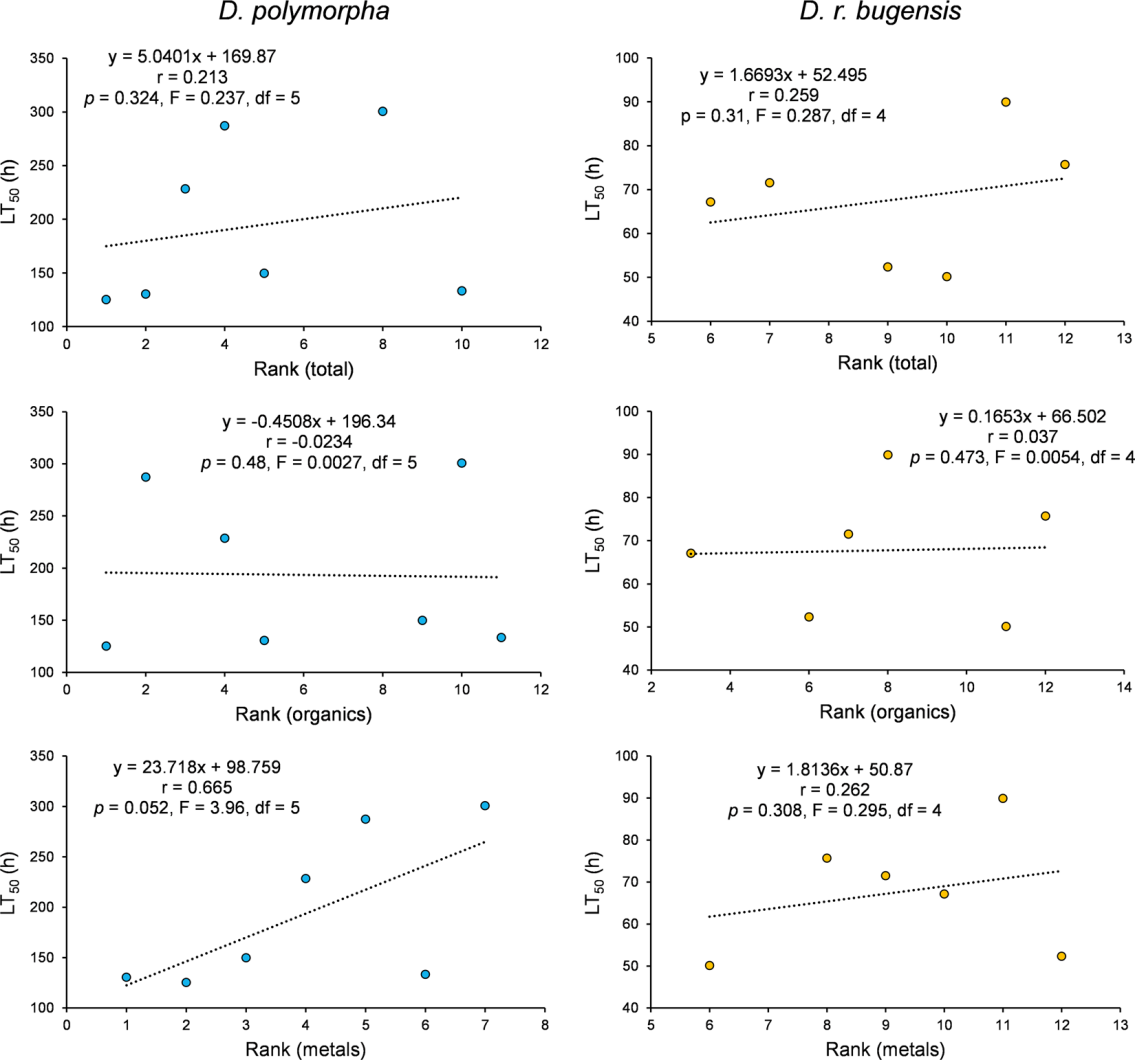
Simple linear regressions between LT_50_ and global contamination (rank) on sampling sites, for the two species separately (D. polymorpha on the left, n = 7; D. r. bugensis on the right, n = 6) and for the three rank types (total, organics and metals). For each regression, equation, correlation coefficient (r), p-value (p), F-statistic (F) and degree of freedom (df) of the model are mentioned.

Since the whole environment can influence tolerance, we have extended correlation analysis to all the measured contaminants. To evaluate a possible influence of site contamination on tolerance, we correlate global contamination of sampling stations and survival of the populations using linear regression. The concentrations of contaminants measured *in situ* are given in Supplementary Table I. A contamination index is also reported for each site; it was calculated using a rank classification, considering all the contaminants (rank total), organic pollutants only (rank organics) or metals only (rank metals). Sites are classified from the least (1) to the most contaminated ones (12). We then tried to correlate mean LT_50_ with these calculated ranks, symbolizing global contamination on each site (Fig. 3). Considering the global contamination or only the organic or metallic ones, no significant correlation was observed. However, we identified a trend toward higher LT_50_ values along with the concentration of metals in the sediment for *D. polymorpha*, which is a little bit higher than the significance level (*p* = 0.052, R² = 0.44, n = 7). The latter result supports the hypothesis that the chronic exposure of *D. polymorpha* to metals may influence its tolerance to acute Ni exposure.

### Physiological mechanisms and differential sensitivities

**Figure 4:**
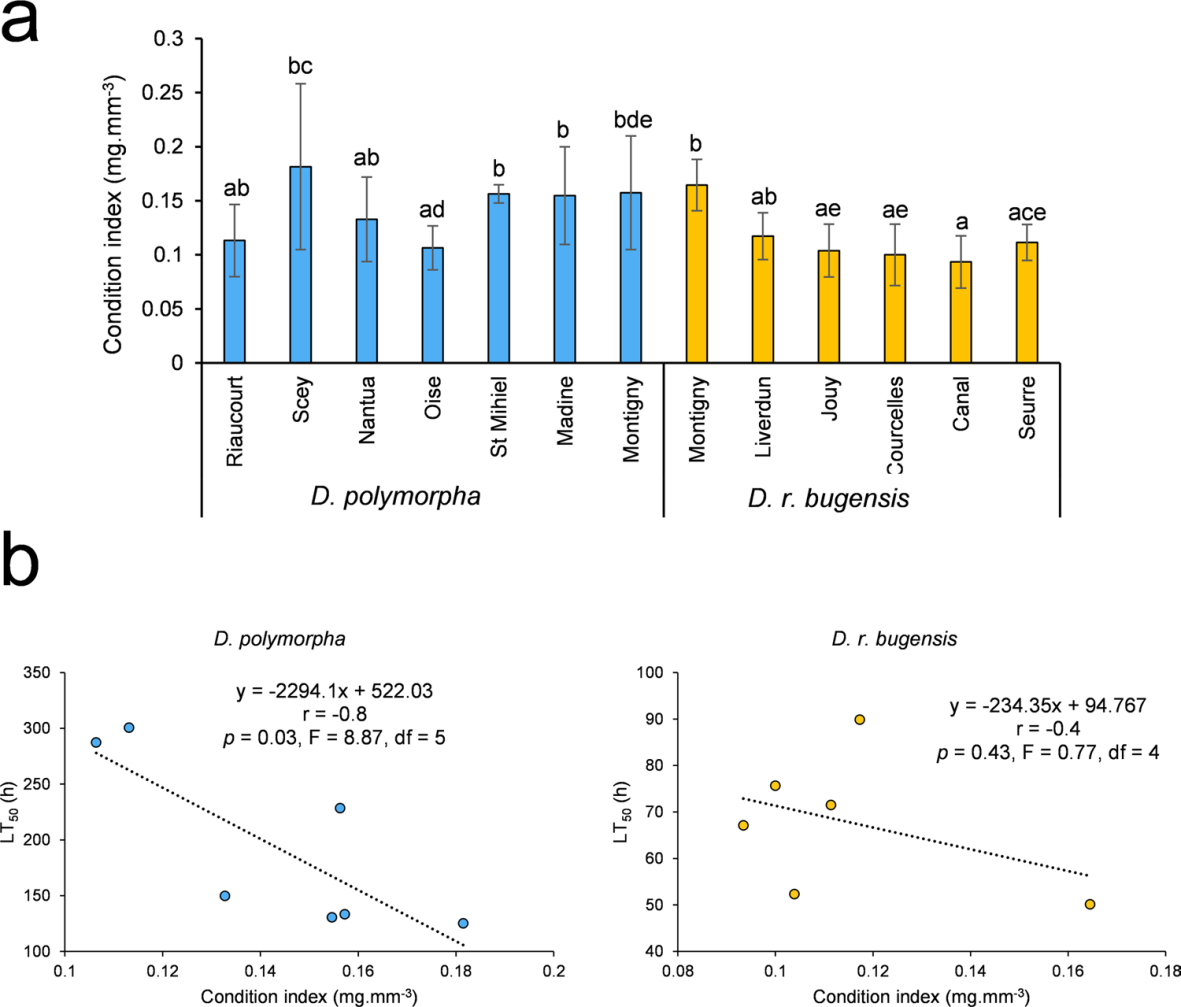
(**a**) Condition index for the different field populations of D. polymorpha and D. r. bugensis (mean ± SD, n = 12). Differences were tested using a Kruskal-Wallis test. (**b**) Simple linear regressions between LT_50_ and condition index for the two species separately (D. polymorpha on the left, n = 7; D. r. bugensis on the right, n = 6). For each regression, equation, correlation coefficient (r), p-value (p), F-statistic (F) and degree of freedom (df) of the model are mentioned.

Condition indices are given in Fig. 4a. Results indicate that, despite some differences, all the populations were of the same health status before starting the experiment. We found no correlation between the mean condition index in the field and the mean LT_50_ (*p* = 0.47, R² = 0, n = 13). However, when looking at species separately again (Fig. 4b), there was a correlation for *D. polymorpha* populations (*p* = 0.03, R² = 0.64, n = 7) but not for *D. r. bugensis* ones (*p* = 0.43, R² = 0.16, n = 6). Surprisingly, the correlation between condition index and LT_50_ is negative, meaning that the lower the condition index is in the field, the better the population tolerates Ni.

**Figure 5:**
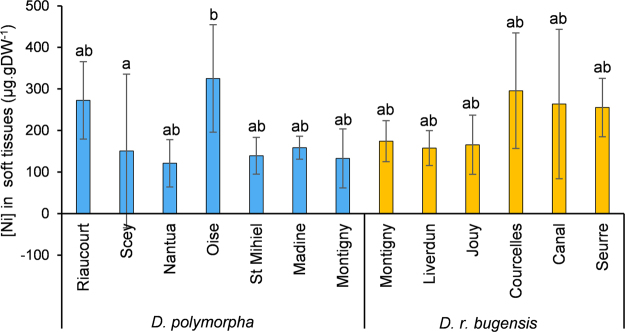
Nickel concentrations in soft tissues after LT_50_ experiment (mean ± SD, n = 5).

Nickel bioaccumulation was measured throughout the experiment (see the experimental design in the methods section). Internal concentrations in Ni after exposure for each population are reported in Fig. 5. The highest mean Ni concentration in controls was 6.4 µg.gDW^−1^ for the Riaucourt population, thus values for controls are not represented here. ANOVA showed that Ni concentrations in soft tissues are different among populations (*p* = 0.004, F = 2.98, df = 11, df_res_ = 52, n = 5) and between species (*p* = 0.039, F = 4.49, df = 1, df_res_ = 52, n = 35 for *D. polymorpha* and 30 for *D. r. bugensis*). Within species, the only inter-populational difference evidenced by the post-hoc Tukey test was between the Scey and Oise *D. polymorpha* populations.

Since we get the exact time of death for each individual, we tried to correlate it with Ni accumulation. A simple linear regression evidenced that Ni internal concentration and time of death were correlated (*p* = 2.87·10^−4^, R² = 0.19, n = 65) meaning that bioaccumulation of Ni increases with exposure time (the later they die, the more they accumulate). If we considered species separately, this positive correlation is true for both species, *D. polymorpha* (*p* = 4.32·10^−7^, R² = 0.54, n = 35) and *D. r. bugensis* (*p* = 5.13·10^−4^, R² = 0.36, n = 30).

We also observed a positive correlation between Ni accumulation and LT_50_ for *D. polymorpha* populations (*p* = 1.87·10^−2^, R² = 0.7, n = 7) but not for *D. r. bugensis* ones (*p* = 0.68, R² = 0.05, n = 6).

### Biomarkers reflect Ni tolerance

**Figure 6:**
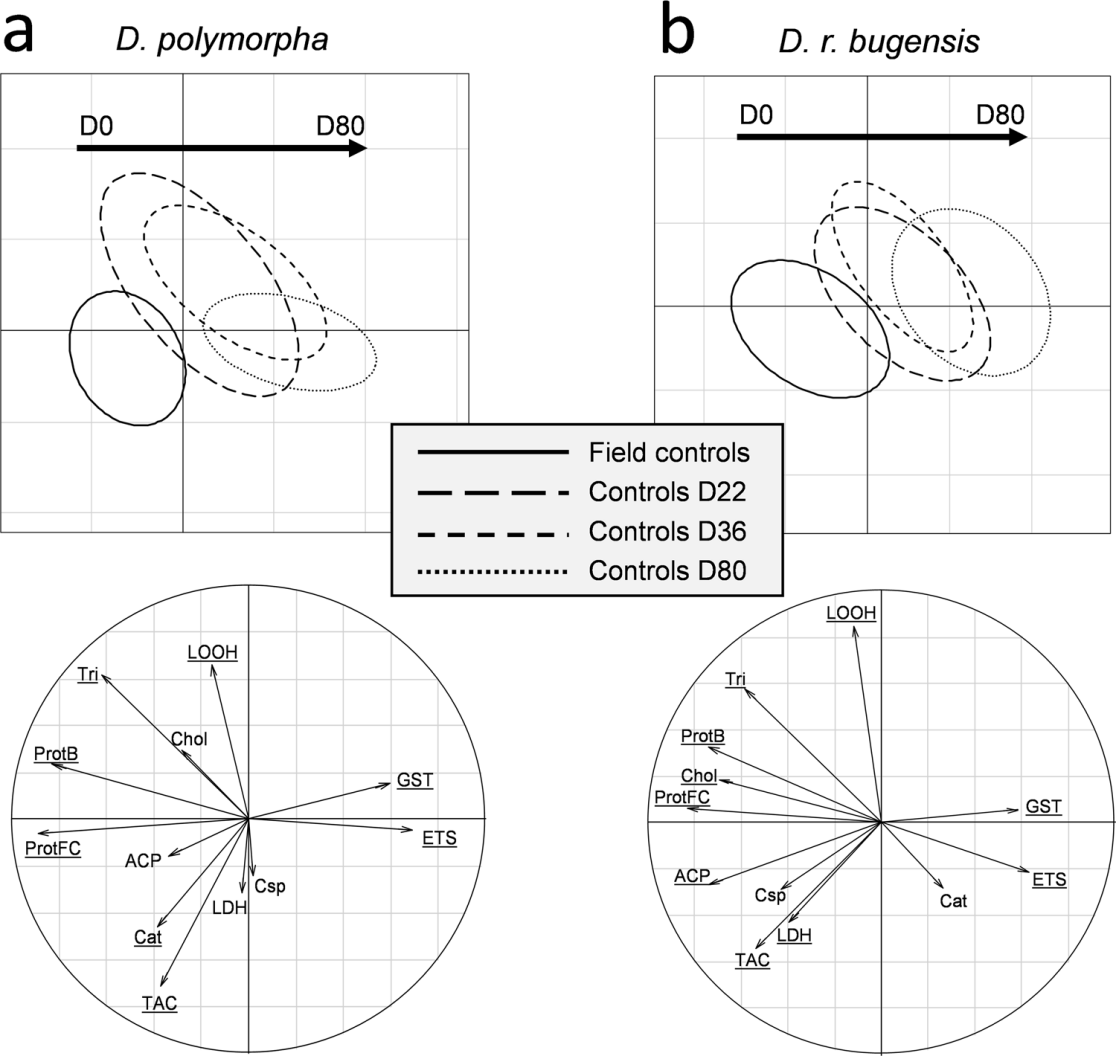
PLS-DA representing biomarker responses in the different controls along the experimentation. Responses of the two species are separated with D. polymorpha on the left (6a) and D. r. bugensis on the right (6b). For a given species, responses of all populations are confounded. Underlined biomarkers in the correlation circles correspond to the VIP (variable importance in the projection) that were higher than 0.8.

First, we looked only at controls, to examine the response pattern in non-exposed mussels (Fig. 6). There is an evolution of biomarker responses from the field to 80 day-exposure conditions in the laboratory when all populations are combined. The response pattern for controls is almost the same for the two species. There is a rational decrease of energetic reserves (Prots, Tri, Chol) with time since the mussels were not fed. Antioxidant defences (Cat, TAC) cannot be maintained, explaining an increased lipid peroxidation (LOOH) at the intermediate time (D22 and D36). The only enzymatic mechanisms still activated in controls after 80 days are GST and ETS.

Then, we correlated biomarkers in field controls with LT_50_, to see if the physiological status observed in the field might reflect tolerance. We selected the biomarkers explaining the major part of the LT_50_ variance using a PLS (Tanagra). We kept Chol, ETS, ACP, GST, TAC, Cat and Csp, which explained 78.3% of the variance on the two first axis. Then, we performed a multiple linear regression to correlate these biomarkers with LT_50_ values. The model evidenced a correlation between biomarker responses in the field and LT_50_ (*p* = 1.39·10^−2^, R² = 0.93, n = 13). Populations with high Cat, ETS and TAC responses seem to better tolerate an acute exposure to Ni. However, this finding might be species-specific, since the higher responses for these biomarkers were in *D. polymorpha* populations.

**Figure 7:**
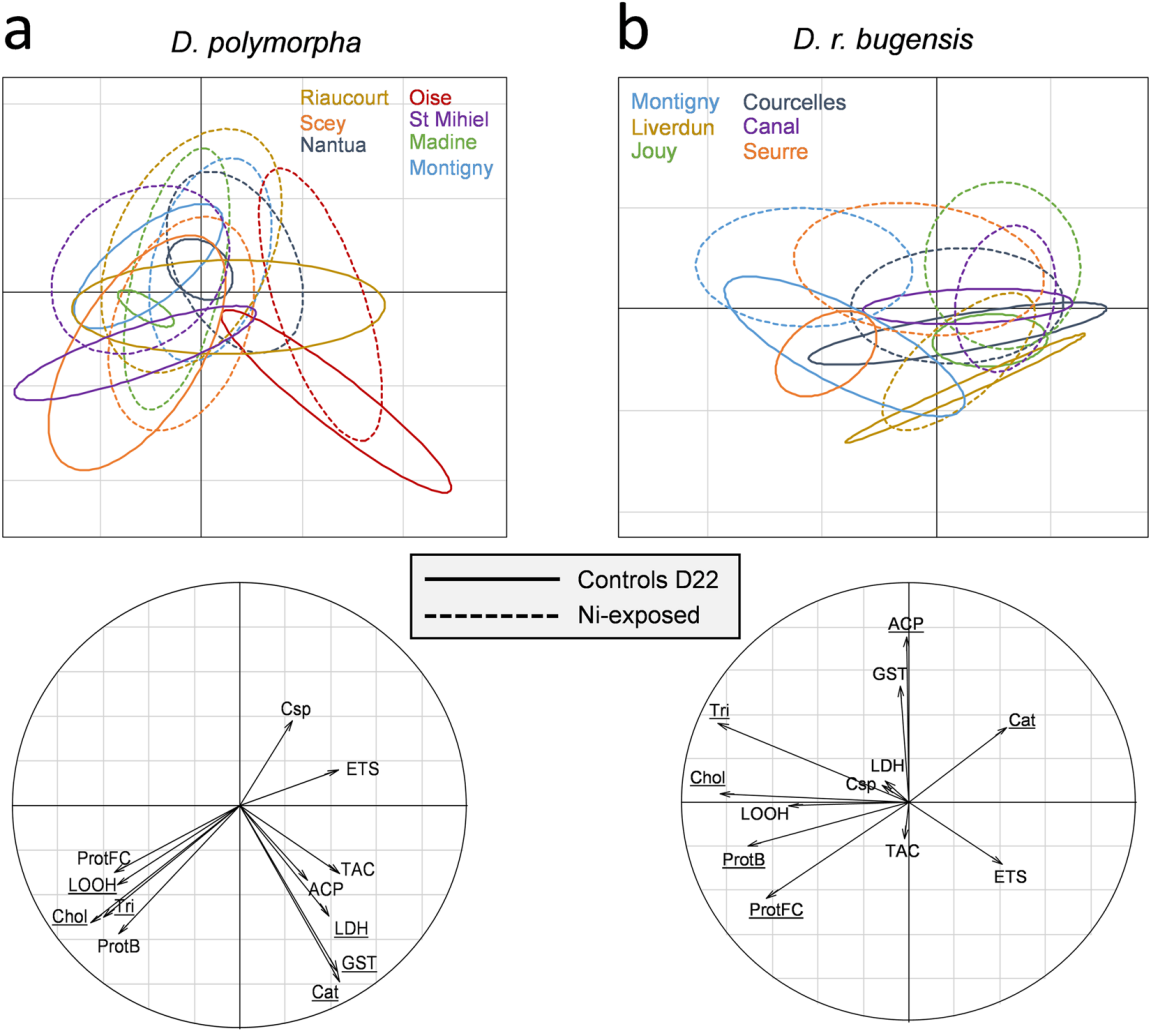
PLS-DA representing biomarker responses in the experimental controls (straight lines, n = 4) and Ni-exposed (dotted lines, n = 10) populations. Responses of the two species are separated with D. polymorpha on the left (7a) and D. r. bugensis on the right (7b). Underlined biomarkers in the correlation circles correspond to the VIP (variable importance in the projection) that were higher than 0.8.

To see how exposure has affected biomarker responses, we compared them between Ni-exposed and experimental control (D22) mussels. On the whole dataset, a MANOVA evidenced a significant effect of species (*p* < 2.2·10^−16^, df = 1, df_res_ = 158, F = 27.56), exposure (*p* = 1.63·10^−6^, df = 1, df_res_ = 158, F = 4.73) and population (*p* = 9.42·10^−16^, df = 11, df_res_ = 158, F = 2.43) on biomarker responses, but there was no interactive effect between these factors. Then, responses of the two species were examined separately under PLS-DA (Fig. 7).

For *D. polymorpha* (Fig. 7a), there was a significant effect of exposure (*p* = 0.026, df = 1, df_res_ = 86, F = 2.11) and population (*p* = 2.28·10^−7^, df = 6, df_res_ = 86, F = 2.25) on biomarker responses, but no interactive effect of these factors. For *D. r. bugensis* (Fig. 7b), these two factors also act significantly (exposure: *p* = 2.52·10^−5^, df = 1, df_res_ = 72, F = 4.669; population: *p* = 1.43·10^−8^, df = 5, df_res_ = 72, F = 2.68) and interact (*p* = 3.42·10^−2^, df = 5, df_res_ = 72, F = 1.41). We observed a diminution of energetic reserves (Prot, Tri, Chol) between controls and Ni-exposed mussels. ETS and LOOH were higher in controls than in Ni-exposed mussels for both species. TAC and LDH activity were maintained in Ni-exposed mussels of both species. GST activity was reduced for *D. polymorpha*, but maintained for *D. r. bugensis* during exposure. ACP was induced in Ni-exposed mussels. Cat activity was reduced in Ni-exposed mussels. The main difference concerned Csp activity, which was highly activated in *D. polymorpha*, but not at all in *D. r. bugensis* populations.

**Figure 8:**
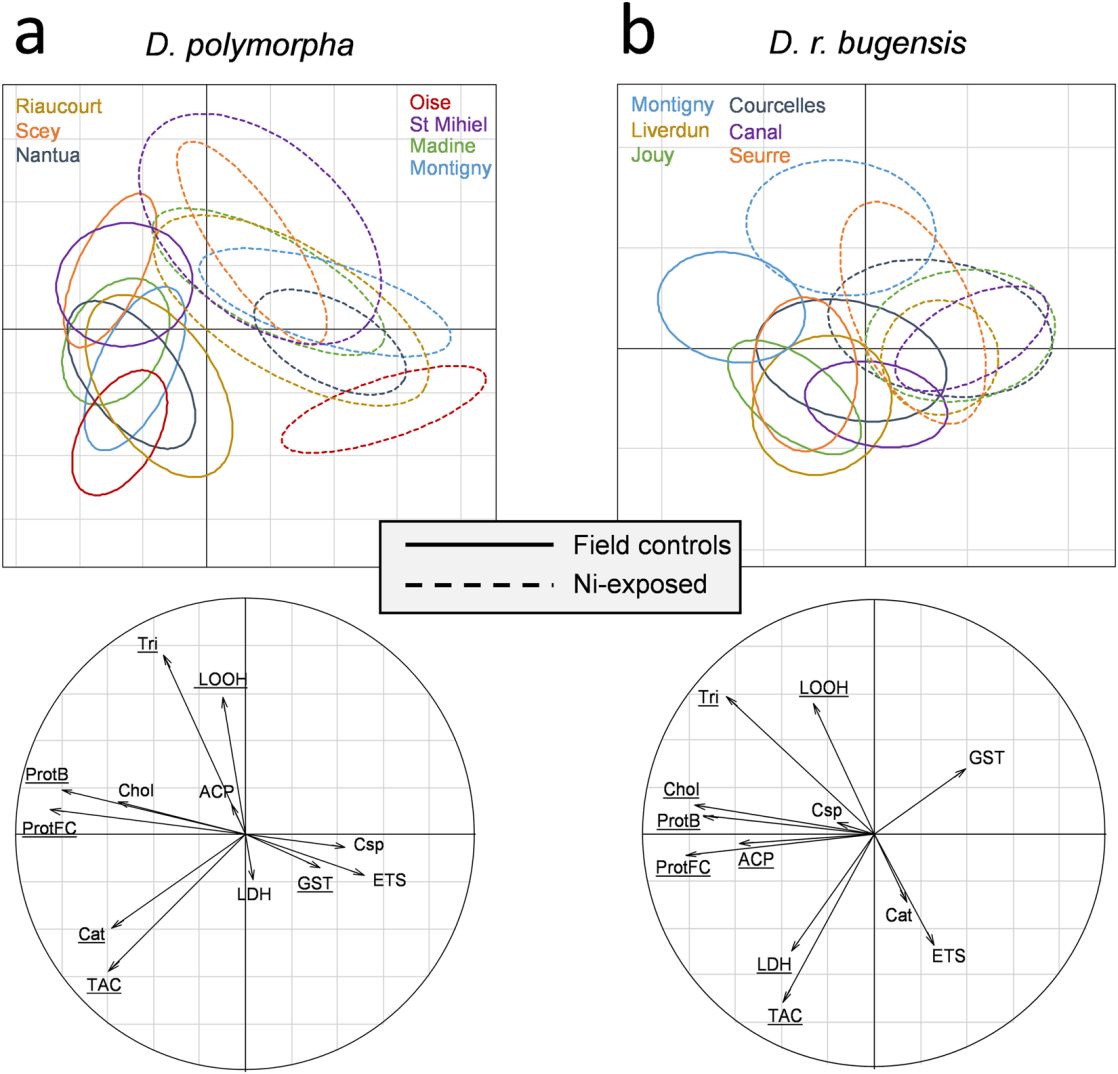
PLS-DA representing biomarker responses in the field controls (straight lines) and Ni-exposed (dotted lines) populations. Responses of the two species are separated with D. polymorpha on the left (**a**) and D. r. bugensis on the right (**b**). Underlined biomarkers in the correlation circles correspond to the VIP (variable importance in the projection) that were higher than 0.8.

Several findings appeared when we compared biomarker responses in field controls and Ni-exposed mussels, to evaluate the effect of acute exposure on organisms (Fig. 8). On the whole dataset, a MANOVA evidenced a significant effect of both species (*p* < 2.2·10^−16^, df = 1, df_res_ = 260, F = 62.37), exposure (*p* < 2.2·10^−16^, df = 1, df_res_ = 260, F = 53.1) and population (*p* < 2.2·10^−16^, df = 11, df_res_ = 260, F = 3.18) on biomarker responses. First, we can see the impact of acute Ni-exposure on organisms with a clear separation of field controls (on the bottom left) and Ni-exposed (on the top right), associated with modifications in biomarker responses for both species.

Second, we can see that *D. polymorpha* populations are more heterogeneous than *D. r. bugensis*, both in the field and after Ni-exposure, since their ellipses are more spread out. In both species, there is no convergence of the responses after exposure, each population maintaining its own range of response.

Concerning *D. polymorpha* (Fig. 8a), there was a significant effect of exposure (*p* < 2.2·10^−16^, df = 1, df_res_ = 140, F = 49), population (*p* = 7.54·10^−15^, df = 6, df_res_ = 140, F = 3.11) and an interactive effect (*p* = 5.47·10^–3^, df = 6, df_res_ = 140, F = 0.71) on biomarker responses. Populations living in the most contaminated sites (Riaucourt, Oise, Montigny and Nantua) are at the bottom of the plot. They differ from the other populations by a higher antioxidative capacity (Cat, TAC) in the field, which disappears along the exposure for the benefit of other mechanisms (increased GST, ETS and Csp activities).

Concerning *D. r. bugensis* (Fig. 8b), there was also a significant effect of exposure (*p* < 2.2·10^−16^, df = 1, df_res_ = 120, F = 18.7), population (*p* = 3.38·10^−14^, df = 5, df_res_ = 120, F = 3.38) and their interaction (*p* = 3.81·10^–3^, df = 5, df_res_ = 120, F = 1.6) on biomarker responses. The response of all populations are close, except for the Montigny one, even after exposure to Ni. This site is not the most polluted, but the population seems to suffer from oxidative stress, with high levels of LOOH and lowered defences. This distress was maintained during the exposure.

Even if GST and ETS seem to be higher in Ni-exposed organisms of both species, the Csp activity is only maintained for *D. polymorpha*. Maybe this species is still able to perform apoptosis to destroy damaged cells, whereas *D. r. bugensis* has surrendered and their mechanisms were completely overwhelmed.

**Figure 9:**
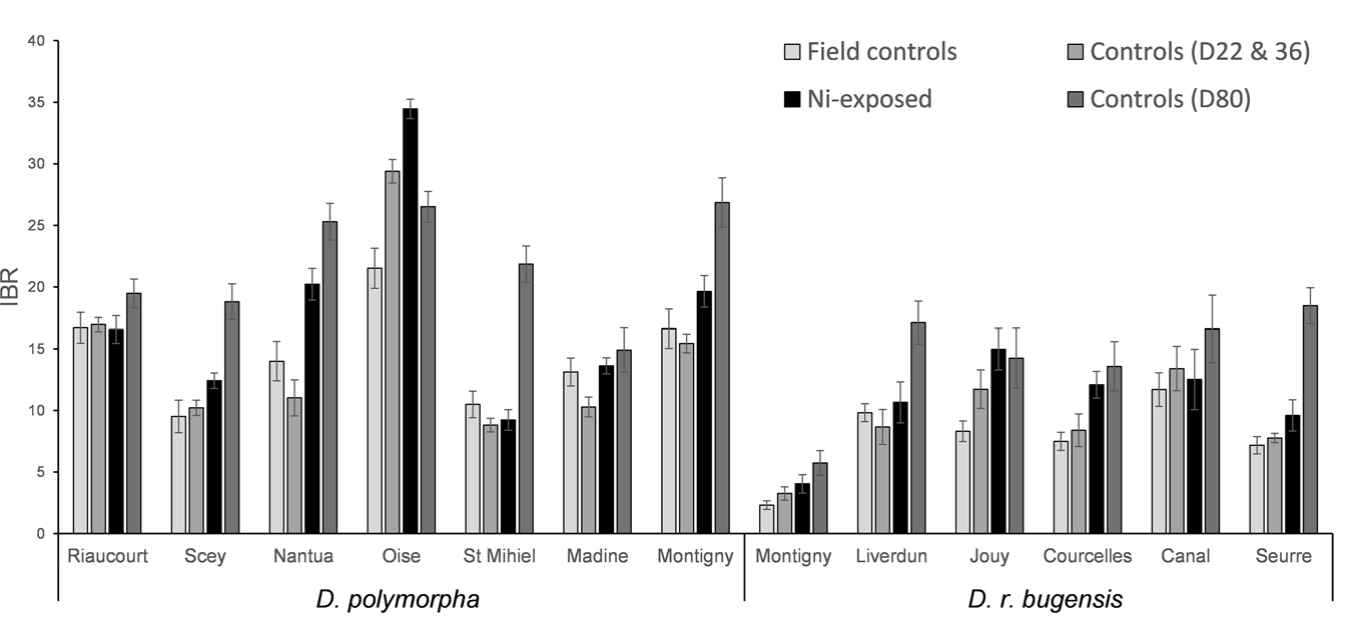
Integrated biomarker responses for the different experimental groups (field controls, n = 12; controls D22 & D36, n = 8; Ni-exposed, n = 10; controls D80, n = 4).

Integrated biomarker response (IBR) was calculated for the different experimental groups (Fig. 9), to illustrate the general response of each population in a given condition (field controls, controls D22 and D36, Ni-exposed, controls D80). There is a general increase in IBR values from the field to the end of the experiment, for both species. IBR values are generally higher for *D. polymorpha* populations, either in the field or in laboratory.

We also tried to correlate IBR values with LT_50_ for each population. We observed a strong correlation between IBR for the field controls and LT_50_ (*p* = 2.1·10^−3^, R² = 0.59, n = 13), which means that the higher the IBR is in the field, the better the population will tolerate Ni.

## Discussion

In this study, LT_50_ assessment evidenced differential tolerances to Ni between dreissenid species and populations, with a higher tolerance to Ni for *D. polymorpha*, and a higher heterogeneity in tolerance for this species. This inter-populational variability has already been shown in the literature for several species^53^, and is often related to the historic exposure of each population. Indeed, we demonstrated that for *D. polymorpha*, tolerance was correlated with Ni concentration in sediments (although this correlation do not integrate the bioavailable portion of Ni *in situ*) and related with metal contamination in general, which was not true for *D. r. bugensis* populations. This link between sediment contamination and tolerance enables to support the cross-tolerance phenomena. Cross-tolerance is the condition in which enhanced tolerance to one toxicant also enhances tolerance to another^20^. In the case of metal tolerance, it has been demonstrated in the laboratory that exposure to cadmium may induce copper tolerance in fish^54^ or lead tolerance in *Daphnia magna*^55^. Cross-tolerance seems to take place for particular pairs of toxic, sharing a chemical structure and mode of action, and depends on the species and the exposed populations. In our case, it is possible that cross-tolerance happened within *D. polymorpha* populations. Indeed, this relationship between sediment contamination and tolerance illustrates the fact that the most metal-exposed populations (Riaucourt, Oise) present the highest LT_50_ values; exposure to other metals might have enhanced individuals’ tolerance to Ni.

Even if it is currently admitted that invasive species have greater genetic variability, upon which natural selection may act to increase their adaptability to new environments^56^, literature is quite contradictory regarding tolerance to environmental stressors in invasive species. On one hand, some studies have shown that invasive species are more tolerant to environmental stressors than native species^57^, and that detoxification and antioxidant systems tend to be more activated in invasive species than in native ones when facing a contamination^58,59^. On the other hand, some have shown the opposite^48,60^. For example, McMahon^60^ has shown that *D. polymorpha* (r-strategy) does not exhibit high resistance or adaptation capacity, but is able to colonize unstable habitats and also to recolonize rapidly after mass extinction, whereas K-strategy native species has to develop an extensive tolerance to maintain. Invasive species are often associated with an r-strategy, characterized by several traits: rapid increase in population density, fast individual growth, early sexual maturity, high fecundity and long distance dispersal. When comparing dreissenid species, it is known that *D. r. bugensis* has a greater growth rate and a better assimilation efficiency, an earlier reproduction period, and seems to be a stronger competitor when both species are present^37,38,61^. These findings, coupled with our results, may indicate that *D. polymorpha*, which is established in Western Europe for a longer time, might approach a more K-strategy than *D. r. bugensis* that remains in a complete r-strategy.

Several studies have shown that West-European *D. polymorpha* populations, established in the 19^th^ century, present a high genetic differentiation^62,63^. On the contrary, *D. r. bugensis*, which colonized only a decade ago, do not show genetic differentiation, and is not expected to be capable of local adaptation^64,65^. However, De Ventura *et al*.^47^ evidenced that local adaptation to fluctuating environmental parameters can rapidly occur even in newly established populations, and showed that *D. r. bugensis* is better adapted to fluctuating oxygen levels than *D. polymorpha*. This adaptability might thus exist, for both species, regarding physico-chemical parameters. However, adaptation to contaminants has not been proved yet, and our results tend to show that local adaptation to contaminants occurred in *D. polymorpha* while it has not yet been set up in *D. r. bugensis* populations. Our findings reflect the longer invasion history of *D. polymorpha*, which has benefited from more time to differentiate and adapt to local conditions. This might explain the greater variability in tolerance to Ni for tested *D. polymorpha* populations and the higher heterogeneity of biomarker responses observed in the field and after exposure to Ni. Such variability has already been evidenced by Pain-Devin *et al*.^9^ who compared several populations of *D. polymorpha* living in more or less polluted sites. Even if *D. polymorpha* populations have a common origin, genes were selected in each population by local environmental conditions, leading to a differentiation; this phenomenon can be enhanced by genetic drift and restricted gene flow^66^.

Studies on local adaptation of dreissenids have rarely focused on adaptation to contaminants and even less on the related physiological or biochemical processes that confer tolerance and that can be used as biomonitoring tools. To counter this, we also assessed sub-cellular biomarker responses and internal Ni concentration in soft tissues, trying to link tolerance with physiological status.

We evidenced that Ni accumulation increased with exposure time for both species, and that an increased Ni accumulation was correlated with a higher tolerance for *D. polymorpha* populations only. This means that both species accumulated Ni, but *D. polymorpha* was more able to handle this accumulation and to defend itself, whereas *D. r. bugensis* was not. The better ability of *D. polymorpha* to defend itself against accumulated contaminants has already been evidenced; this species tend to induce its defence mechanisms, such as hsp70 in presence of organic compounds^46^ or antioxidative mechanisms in presence of metals^7^, whereas *D. r. bugensis* suffers more from cellular damages (DNA damages or lipid peroxidation, for both experiments respectively).

Beyond a better comprehension of differential tolerance in natural populations, our study also raises new insights concerning the interpretation of biomarker responses. Measuring biomarker responses on dying animals gave an idea of their biochemical status as they were close to death, and enabled a comparison with those measured *in situ*. We observed a decrease in energetic reserves (Prot, Tri, Chol), probably due to the absence of food during the experiment. Since the Ni-exposed mussels on which we measured biomarker responses were almost dying, we can hypothesize that organisms were in physiological distress. This is not surprising considering the high dose of Ni they were faced to (2.5 mg.L^−1^). Classically, ecotoxicological studies carried out to evaluate biochemical and physiological responses are performed at low concentrations of contaminants, close to those observed *in situ*. Here, the main goal was to obtain LT_50_ values, and the high concentration of Ni selected to evaluate this parameter was responsible for this distress. Even if cellular mechanisms in Ni-exposed mussels were greatly disrupted, *D. polymorpha* seemed more able to defend itself, with a higher GST activity and mainly a higher caspase activity, attesting that this species was still able to eliminate damaged cells whereas *D. r. bugensis* was completely overwhelmed. It would thus be interesting to focus on refining biomarker responses. For example by selecting two contrasted populations of both species, and exposing them at a more realistic concentration, with food implementation, to get a more field-representative assessment. Beyond these general trends, we also evidenced an interaction between population and exposure in almost all situations, which underlines that each population gets some specific features when dealing with contamination.

Biomarkers measured in field mussels showed that *D. polymorpha* presents higher levels of Cat, ETS, TAC *in situ* compared to *D. r. bugensis*. Thus, defence mechanisms (and especially antioxidant defences) are more activated for this species and seem to confer a better tolerance when exposed in the laboratory. In accordance with our previous results^7^, antioxidant mechanisms thus seem to be good candidates for biomonitoring through the use of biomarkers. The correlation between these responses and LT_50_ attests that for *D. polymorpha*, high biomarker responses in the field might predict a greater tolerance when exposed to contaminants, while it is more often interpreted as a marker of population at risk.

We also integrated all biomarker responses in a general stress index using IBR. Results evidenced a lower IBR index for *D. r. bugensis* populations (both in the field and in laboratory), which theoretically means that they were less stressed than *D. polymorpha*. However, considering that all the results indicate a higher stress for *D. r. bugensis*, the lower IBR values for this species might just be due to a lower activity of cellular mechanisms used as biomarkers. According to Bocquené *et al*.^67^, an IBR value higher than 9 means that individuals suffer from strong stress. Looking at IBR values for *D. polymorpha* in the field reveals that almost all populations exceed this threshold, but after exposure, it turns out that it seems to be the more tolerant species. Moreover, a high IBR index in the field is correlated with a high LT_50_. Thus, when considering local adaptation in our interpretation of biomarker results, the common framework is questioned, since a high IBR value cannot necessarily be associated with a stress response and does not always mean that the population is in danger, and can even indicate a better ability to cope with pollution.

Tolerance might also depend on life-stage, as shown by Elderkin *et al*.^49^, or on seasonal fluctuations, as shown by Costa *et al*.^68^ who evidenced that tolerance to a molluscidal agent in *D. polymorpha* was lower during summer. A higher filtration rate and a poor health status during summer would reduce energy allocation to defence mechanisms, and may be responsible for this lowered tolerance. Such parameters (e.g. life-stage, season, reproductive status, gender) can influence tolerance and should be taken into account for future environmental monitoring using this species.

In conclusion, this study evidenced that there is a differential inter- and intra-specific tolerance to Ni in the two freshwater bivalves *D. polymorpha* and *D. r. bugensis*. Populations of *D. polymorpha* have a better ability to cope with anthropogenic stressors. This species has a longer invasion history and presents a better adaptability to local environmental conditions, explaining the greater heterogeneity observed between *D. polymorpha* populations. This work also highlighted the need to take into account differential tolerance among populations of a given species to be used in biomonitoring, since it is obvious that different populations of a same species will not respond the same way to stressors. This variability must be considered as a confounding factor when using biomarker responses in biomonitoring. In accordance with our previous results, it also underlined that *D. r. bugensis* populations are less differentiated and more sensitive to contamination. Thus, a thorough understanding of sentinel species and populations is needed to correctly interpret their responses to environmental changes.

## Methods

### Organism collection and biometric data

The 13 bivalve populations were hand-collected in April 2016 by section of their byssus, placed in separated aerated coolers (1 cooler per population), and transferred to the laboratory until LT_50_ experiment. The two species were differentiated according to morphometric parameters^69^ and all specimens were adults (22.5 ± 3.6 and 25.2 ± 4 mm in length for *D. polymorpha* and *D. r. bugensis* respectively).

During sampling, 12 mussels per population were measured (length, width, height of shell, in mm) and soft tissues were weighed in order to calculate an individual condition index according to the following formula^70^:

$${\rm{Condition}}\,{\rm{Index}}\,(\mathrm{CI}\,{\rm{in}}\,{\rm{mg}}{{\rm{.mm}}}^{-{\rm{3}}})={\rm{wet}}\,\mathrm{weight}/({\rm{length}}\ast {\rm{width}}\ast {\rm{height}})$$

Thereafter, the digestive gland of these 12 mussels were dissected *in situ* and frozen in liquid nitrogen, and kept at −80 °C until biomarker measurements. They were used as a field control allowing us to have the originating response.

### Acclimatization

Organisms used for LT_50_ assessment were acclimatized during 3 days at 12 °C, which corresponds to the mean temperature for the different sampling sites (11.9 ± 1.9). Thirty organisms of each population were placed in 10 L aerated aquaria, without food, under natural photoperiod. Water from the field was progressively replaced by spring water (Cristaline®, 1/3 per day; Ca^2+^: 106; Mg^2+^: 4.2; Na^+^: 3.5; K^+^: 1.5; HCO_3_^−^: 272; SO_4_^2−^: 50; Cl; F^−^: 0.9 mg L^−1^). Thereafter, organisms of each population were placed in 600 mL experimental beakers (six beakers per population, with five individuals per beaker) to allow byssal fixation. Beakers were filled with 600 mL of Cristaline® and placed in a water bath to maintain a 12 °C temperature, under natural photoperiod.

### LT_50_ experiment

**Figure 10:**
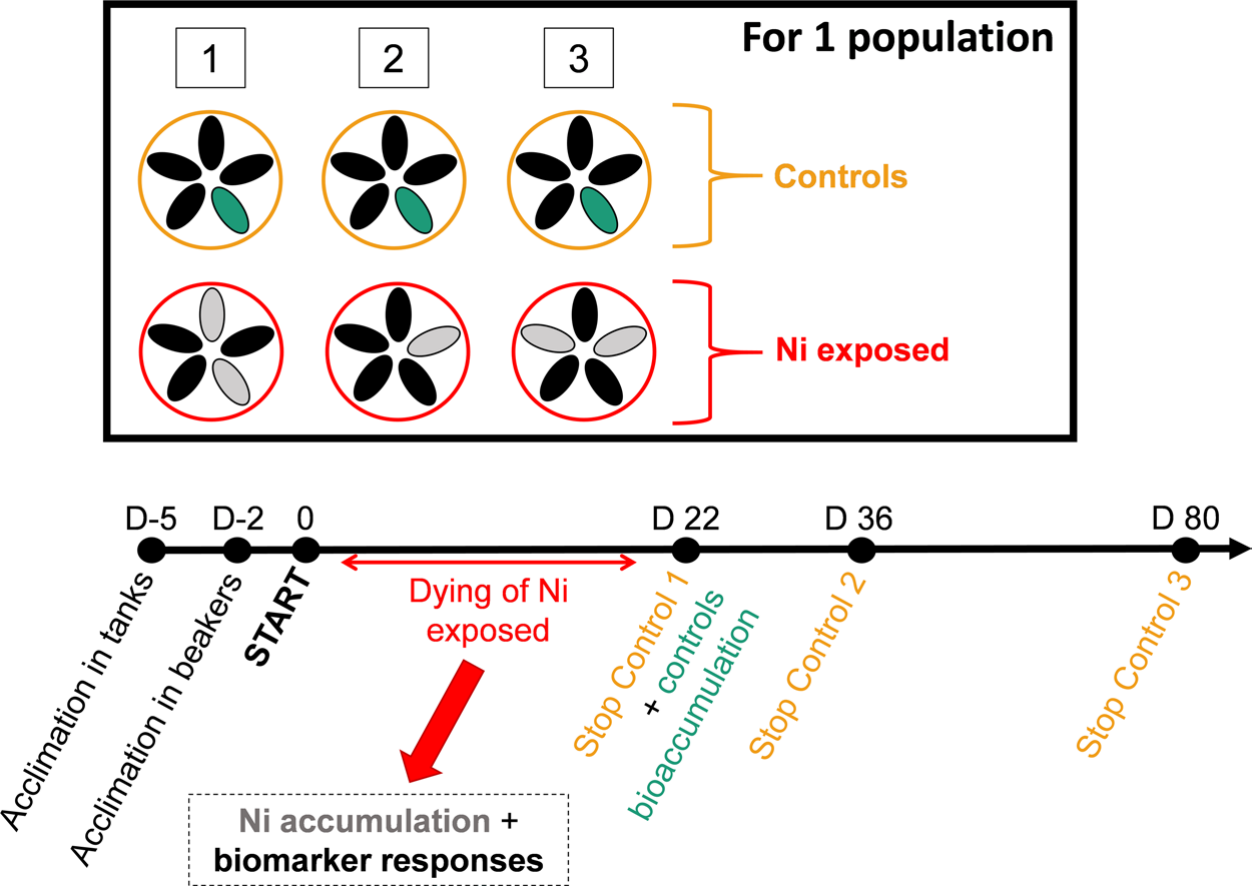
Experimental design.

After acclimatization, for each population, half of the mussels were exposed to nickel (15 organisms, distributed in 3 beakers, 5 mussels per beaker) at 2.5 mg.L^−1^ (Fig. 10). This concentration was chosen according to previous experiments (with several metals at several concentrations; unpublished data) because it enabled the obtainment of LT_50_ values in short exposure time, allowing the effects of starvation to remain negligible. A stock solution of nickel chloride (Cl_2_Ni · 6H_2_O, Nickel(II)chloride hexahydrate AnalR NORMAPUR VWR Ref 25851236) at 500 mg.L^−1^ (expressed as total ion concentration) was prepared and acidified to 1% with nitric acid. Nominal test concentration was made by diluting Ni stock solution.

Experimental beaker water was not aerated to limit metal adsorption upon glass walls. All media were prepared with Cristaline® and were renewed every 48 h, both to ensure a semi-static exposure, and to avoid a drop in oxygen concentrations within the beakers. The 15 remaining organisms were exposed only to Cristaline® and used as controls. Beakers were placed in 12 °C water bath. Nickel concentrations within the beakers were checked before every renewal and were 2.44 ± 0.05 mg.L^−1^. Five mL of exposure media were sampled in each diluted Ni solution and Ni concentration was measured using flame atomic absorption spectrophotometry (Perkin-ELMER Aanalyst 100).

### Mortality check-up

Mortality was checked twice a day (in the morning and at the end of the afternoon). Mussels were considered alive when they were closed, when they rapidly closed their valves after prodding, and when they exhibited filtration activity (siphons out). Mussels that were too slow, or not fully closed, were considered dying and were removed from the beakers.

### Use of organisms

All Ni-exposed mussels died before controls. Five mussels were randomly selected for bioaccumulation measurement, and the digestive gland of ten remaining mussels were used for biomarker measurement.

The first control beaker was stopped after 22 days, just after the last Ni-exposed mussel died, in order to compare biomarker responses between controls and exposed organisms. At that time, 1 mussel per control beaker was picked to measure bioaccumulation. The two remaining beakers, containing then 4 mussels were left. The second was stopped after 36 days, and the last one after 80 days. This prolongation for the controls enabled to (i) ensure that laboratory conditions were not responsible for organism’s death, and (ii) to observe the evolution of biomarker responses on the long term. Even if mussels were unfed, a development of biofilm was observed after 1 month in the control beakers, probably due to algae that remained on the shells.

### Biomarker measurements

Biomarker responses were measured using an automated spectrophotometer (Konelab 20-XTi, ThermoScientific). This device operates on very small volumes and enables the measurement of the whole set of biomarkers on each digestive gland, which is considered as the most metabolically active organ in mussels^71^.

Digestive glands were defrosted, weighed and crushed in phosphate buffer (50 mM, pH 7.6 supplemented with 1 mM phenylmethylsulfonyl fluoride (PMSF) and 1 mM L-serine borate mixture as protease inhibitors) at a 4/1 (v/w) ratio. Homogenates were centrifuged at 250 × *g* for 5 min at 4 °C (crude extract). Homogenates were used for the assays immediately after their preparation. One part of this fraction was used to evaluate triglyceride (Tri), cholesterol (Chol), lipid hydroperoxide concentration (LOOH), electron transport system (ETS) and acid phosphatase (ACP) activities. The other part underwent a differential centrifugation (1000 × *g* for 20 min at 4 °C; the supernatant was recovered and centrifuged again at 20,000 × *g* for 50 min at 4 °C). The final supernatant corresponding to the cytosolic fraction was used to measure total antioxidant capacity (TAC), glutathione-S-tranferase (GST), lactate dehydrogenase (LDH), caspase-3 (Csp) and catalase (Cat) activities. Proteins were quantified on both fractions (crude extract (ProtB) and cytosolic fraction (ProtFC)) by the Pyrogallol red colorimetric method, using Scal bovine-based serum as a reference. Biomarker responses were measured following classical methods, adapted for automated measurements in our spectrophotometer. The detailed protocols are available in Garaud *et al*.^6^, except for Csp and Cat which were slightly modified for microplate measurement in a spectrofluorimeter (SAFAS Xenius, Monaco).

For Cat measurement, cytosolic fraction (5 µL) was diluted in homogenizing buffer (130 µL) and placed in a UV-clear transparent microplate. An automated injector added 65 µL H_2_O_2_ at 92.3 mM, the plate was agitated and the decrease in absorption was followed at 240 nm during 30 seconds. Results are expressed in mmol H_2_O_2_. g prot^−1^.min^−1^.

For Csp measurement, 20 µL of cytosolic fraction were placed in a dark microplate, and 120 µL of reagent (Tris 13.4 mM containing 350 µM DEVD-AFC, 5.6 mM DTT, 2.2 mM EDTA) were added. The fluorescence kinetic of AFC, cleaved by the caspases, was measured at 400 nm (emission) and 505 nm (excitation) during 30 min. Results are expressed as µmol AFC.g prot^−1^.h^−1^, relative to a standard of AFC (from 50 to 0 µM AFC).

### Metal internal concentration analysis and sediment contamination

Total soft tissues (without byssus) were used for the measurement of internal concentration in Ni. Mussels were defrosted, dried in an oven at 60 °C for 24 h, weighed and digested in 5 mL 69% HNO_3_ for 48 h at 65 °C. The amount of Ni was determined by flame atomic absorption spectrophotometry (Perkin-ELMER Aanalyst 100). Metal concentrations in mussels are expressed as µg g^−1^ dry weight (DW) tissue. To ensure a correct measurement, we used a standard mussel tissue (ERM^®^-CE278K) and we measured a concentration of 0.8 µg.gDW^−1^ in Ni that falls within the 0.54–0.84 µg.gDW^−1^ range specified for the standard, and attests the reliability of our measurements.

Sediments were sampled in our 12 studied sites. We made 3 sampling points per site, mixed them, and sent 500 g for metals, PCBs and HAPs analysis to La Drome Laboratoire (Cofrac accreditation n° 1-0852). Sediments were mineralized according to EPA 3052 method (using hydrofluoric and nitric acids). Total contaminant concentrations were measured on the <2 mm fraction, using gas chromatography-mass spectroscopy (GC-MS) for HAP and PCB (French norm XP X 33-012), inductively coupled plasma atomic emission spectroscopy (ICP-AES) for Al, Fe, Mn, Zn (EN ISO 11885), inductively coupled plasma mass spectroscopy (ICP MS) for As, Cd, Cr, Co, Cu, Ni, Pb (EN ISO 17294-2) and cold vapour atomic fluorescence spectrophotometry for Hg (EN ISO 17852). A rank test was used to build a contamination gradient of sites, and attribute to each one a contamination index. We transformed the quantitative variable associated with each contaminant into a discrete variable representing the rank of each station within the set of studied stations. The ranks are then summed for all contaminants (rank total), organic pollutants only (rank organics) or metals only (rank metals). Sites are classified from the least (1) to the most contaminated (12).

### Statistical analysis

Statistical analysis was made using R software (R Development Core Team, version 3.3.0) and Tanagra (version 1.4). Non-linear regressions were performed using the REGTOX macro under Excel^TM^ (version 7.0.7, Hill model) to determine the time inducing 50% mortality in tested populations (LT_50_, lethal time for 50% of the population). Normality and homoscedasticity were tested respectively with Shapiro and Bartlett tests, followed by analysis of variance (ANOVA, MANOVA or Kruskal-Wallis, depending on previous tests). Inter-condition differences were evaluated through post-hoc Tukey or KruskalMC tests. Considering the low number of replicates (3) used to calculate LT_50_ values for each population, power analysis were performed using G Power (version 3.1) for the observed significant differences; values were always above the 0.8 threshold, which is the classical admitted limit for a good quality power.

Correlations were tested using simple and multiple linear regression. Results of ANOVA statistics for linear regression analysis are given as proportion of variance explained (r squared, R²) and *p* value of the F-test. Some data were transformed by log10, to avoid the influence of high values.

Partial least square discriminant analysis (PLS-DA) was performed to assess which biomarker responses were able to discriminate experimental groups, after selecting the influent biomarkers using PLS function under Tanagra. Then the PLS-DA was performed and the significant biomarkers (variable importance in the projection, VIP > 0.8) are underlined on the graphics.

A more synthetic representation of biomarker responses is also provided with IBR (Integrated Biomarker Response) calculation^72^, an index commonly used in ecotoxicology to synthetize multibiomarker studies.

For all analysis, a threshold of *p* ≤ 0.05 is considered significant and degrees of freedom (df) and residual degrees of freedom (df_res_) are reported. Data on the graphics are presented as mean ± standard deviation (SD).

### Data Availability Statement

The data that support the findings of this study are available from the corresponding author upon reasonable request.

## Electronic supplementary material

Supplementary information 
