# Supplementary material for: Differential tolerance to nickel between Dreissena polymorpha and Dreissena rostriformis bugensis populations
Source: Sci Rep. 2018 Jan 15;8:700. doi: 10.1038/s41598-018-19228-x (PMC5768691; doi:10.1038/s41598-018-19228-x)
Supplement: Supplementary file 1 — Supplementary information [file 41598_2018_19228_MOESM1_ESM.doc]

**Title**

Differential tolerance to nickel between *Dreissena polymorpha* and *Dreissena rostriformis bugensis* populations

**Authors**

Marine Potet*^1^, Laure Giambérini^1^, Sandrine Pain-Devin^1^, Fanny Louis^1^, Carole Bertrand^1^, Simon Devin^1^.

^1^ Université de Lorraine, CNRS UMR 7360, Laboratoire Interdisciplinaire des Environnements Continentaux (LIEC), Campus Bridoux, rue du Général Delestraint, 57070 Metz, France

* Correspondence to: marine.potet@univ-lorraine.fr

Supplementary Table I

Supplementary Table I: Contaminant concentrations in the sediments for the 12 sampling sites. Results are expressed relative to dry matter (DM). Sites are classified from the least (1) to the most contaminated (12), considering all the contaminants (rank total), organic pollutants only (rank organics) or metals only (rank metals). Colours indicate the contamination level according to the rank (green: four least contaminated; yellow: four intermediately contaminated; red: four most contaminated).
